# Application of On-Line nanoLC-IT-TOF in the Identification of Serum β-Catenin Complex in Mice Scald Model

**DOI:** 10.1371/journal.pone.0046530

**Published:** 2012-10-09

**Authors:** Cheng-cai Huang, Shi-hai Yan, Dan Chen, Bi-cheng Chen, Ning-wei Zhao

**Affiliations:** 1 Life Science & Clinical Medicine Dept., Shimadzu (China) Co., Ltd., Shanghai, China; 2 Department of Pharmacology, Jiangsu Provincial Hospital of Traditional Chinese Medicine, Nanjing, China; 3 State Key Laboratory of Pharmaceutical Biotechnology, School of Life Science, Nanjing University, Nanjing, China; 4 Department of Surgery, The First Affiliated Hospital of Wenzhou Medical College, Wenzhou, China; 5 School of Biotechnology, Royal Institute of Technology (KTH), Stockholm, Sweden; National Cancer Center, Japan

## Abstract

Severe burn shock remains an unresolved clinical problem with an urgent need to explore novel therapeutic treatments. Intracellular β-catenin, through interaction with other proteins, has been reported to be able to regulate the size of cutaneous wounds. Higher expression of β-catenin is associated with larger sized wounds. However, the identification of serum β-catenin complex is difficult and has been rarely reported. The exploitation of more binding partners can contribute to uncovering the exact mechanisms behind serum β-catenin mediated biological effects. Here, we describe a method that consists of immunoprecipitation, SDS-PAGE, in-gel digestion, and nanoLC coupled to LCMS-IT-TOF for the investigation of serum β-catenin complex in mice scald model. Among selected gel bands obtained from the protein gels, a total of 31 peptides were identified and sequenced with high statistical significance (p<0.01). Three proteins (alpha-2-marcoglobulin, serine protease inhibitor A3K, and serine protease inhibitor A1A) were identified and validated with high reliability and high reproducibility. It was inferred that these proteins might interact with serum β-catenin, which could affect the wound healing resulting from burn shock. Our study demonstrated that the on-line coupling of nano-LC with a LCMS-IT-TOF mass spectrometer was capable of sensitive and automated characterization of the serum β-catenin complex in mice scald model.

## Introduction

β-catenin usually forms complexes with other proteins to regulate cell growth and adhesion between cells, which are required for the creation and maintenance of epithelial cell layers. β-catenin also anchors the actin cytoskeleton and may be responsible for transmitting the signal of contact inhibition that causes cells to stop dividing once the epithelial sheet is complete [1]. Recent evidence has suggested that β-catenin plays a significant role in various aspects of liver biology including liver development (both embryonic and postnatal), liver regeneration following partial hepatectomy, HGF-induced hepatomegaly, liver zonation, as well as the pathogenesis of liver cancer, in which β-catenin signals act through the interaction with other proteins [2]. β-catenin contains armadillo repeats and is able to interact with other proteins. Inside cells, β-catenin can be found in complexes with cadherins, transcription factors, and other proteins such as axin (an important component of the Wnt signaling pathway), and galectin-3 (β-galactoside-binding protein). The ability of β-catenin to interact with other proteins is regulated by tyrosine kinases [3] and serine kinases, such as GSK-3 [4]. When β-catenin is not assembled in complexes with cadherins, it can constitute a complex with axin. While bound to axin, β-catenin can be phosphorylated by GSK-3, which transmits a signal for the rapid ubiquitin-dependent degradation of β-catenin by proteosomes. Various signals (such as Wnt) can inhibit GSK-3-mediated phosphorylation of β-catenin [5], allowing β-catenin to translocate into the cell nucleus, bind to transcription factors, and regulate gene transcription. β-catenin can be phosphorylated by other kinases such as protein kinase A (PKA). Also, β-catenin phosphorylation has been associated with reduced degradation of β-catenin, increased levels of β-catenin in the nucleus and the interaction of β-catenin with TCF family transcription factors, which can regulate gene expression [6]. Serum β-catenin has been regarded as a potential marker for genotype 4/hepatitis C-associated hepatocellular carcinoma [7]. β-catenin was partially co-localized and co-isolated with the raft-associated membrane protein caveolin-1 and glycosylphosphatidylinositol-anchored protein CD59, suggesting its potential excretion into the extracellular milieu (serum) through exosome/prostasome associated pathways [8]. However, surprisingly, it was discovered that the extracellular β-catenin was not rapidly degraded; on the contrary, it interacted with some unknown factors in our experiments. Thus, the identification of serum β-catenin complex attracted our attentions, and we felt the exploitation of more binding partners could contribute to uncovering the exact mechanisms behind serum β-catenin mediated biological effects.

Mass spectrometry (MS) has become a major tool for identifying protein complexes and can be used in the analysis of small molecules as well [9]. Most mass spectrometers can be used for tandem mass spectrometry (MS-MS) to obtain structural information by the fragment ions. Ion trap instruments can operate multiple stages of MS-MS (MSn) analysis to provide additional structural information that help with in-depth study on post-translational modifications such as glycosylation. The most popular protein experiments aimed at protein identification and expression involve online nano-flow liquid chromatography (nanoLC) tandem mass spectrometry (LC-MS-MS), because nano-scaled analysis increases the sensitivity with extremely low sample volume [10]. In the nano flow-rate range, stable solvent delivery is especially important in the performance of nanoLC. Prominence nanoLC (Shimadzu, Kyoto, Japan) equips the unique design - Reflux Flow Control (RFC) system for each pump independently. The flow rate is controlled by feedback from signals from the high - precision nano flow sensor, so that precise flow rates are always assured. Additionally, volume between ports is lower than 25 nL, and peak broadening is virtually zero in the nano flow-rate range, thus the capacity of protein/peptide separation is efficiently maximized with high peak resolution.

Intracellular β-catenin has been reported to be able to regulate the size of cutaneous wounds, with higher expression associated with larger sized wounds [11], [12]. However, the serum β-catenin complex participating in the wound healing process has been rarely reported. In this paper, we describe a method that consists of immunoprecipitation, SDS-PAGE, in-gel digestion, and nanoLC coupled to LCMS-IT-TOF, which contributed to investigating the serum β-catenin complex in mice scald model.

## Materials and Methods

### Sample preparation

Twenty healthy mice were donated by the Nanjing University of Traditional Chinese Medicine (TCM), and they were followed up with stable body status for three weeks. They were then divided into two groups (control group and scald group). Before the onset of shock, the mice were anesthetized with ether inhalation to ameliorate suffering. Next, the lower trunk and lower extremities (35%–40% of total body surface) of the scald group in mice were scalded with 80°C water for 15 s to produce the burn shock. The onset of shock was confirmed by recording a sustaining decline of mean arterial blood pressure (MAP) in 3 h [13]. Thirty minutes later, aliquots of serum specimens were taken around the scalded tissue in the scald group and aliquots of serum specimens were taken around the similar area in the control group with a disposable 1.5-mL low-absorption tube. Samples were frozen at −20°C for further analysis. All relevant animal experiments were approved by the animal study committee of the ethics board of Jiangsu Provincial Hospital of TCM (WHOSIDCER) and were performed in accordance with the guidelines of NIH, USA. The bovine serum albumin (BSA) tryptic digests, which is a complex mixture of peptides produced by trypsin digestion of BSA that was reduced and alkylated with iodoacetamide, was ordered from New England Biolabs and were used as control samples in nanoLC coupled to LCMS-IT-TOF for protein identification. The serum samples (each 1 mL) were subjected to immunoprecipitation.

### Immunoprecipitation

Each serum sample was cleared by centrifugation at 20,000 g for 10 min. The immunoprecipitation of endogenous β-catenin was performed by overnight incubation of the cleared lysates with agarose-conjugated goat anti-β-catenin antibodies (Santa Cruz Biotechnology, catalog number sc-1496) [14], followed by three washes with 1 ml PBS buffer. The immune complexes were boiled in Laemmli buffer and subjected to SDS-PAGE and Western blotting with the desired antibodies.

### SDS-PAGE and Western blotting analysis

The boiled immune complexes were resuspended in 100 µL 1×sample buffer (62.5 mM Tris-HCl, pH 6.8, 10% glycerol, 2% SDS, 0.0025% bromophenol blue and 5% of 2-mercaptoethanol), and were heated at 100°C for 5 min. The samples (15 µL per lane) were run at a constant 120 V for 2 h on an 8%–16% gradient gel. The gel was stained for 1 h at room temperature with Coomassie blue R-250, or transferred to a PVDF membrane and detected by blot analysis with anti-β-catenin antibodies.

### In gel digestion

The luminous density differences of the gel bands between two groups were analyzed by BandScan software, and a one-way ANOVA was used to evaluate the significance of inter-group differences. Contrasted with the control group, three clear gel bands with higher protein expression were selected from the scald group for in-gel digestion, except for the gel bands containing β-catenin protein and anti-β-catenin IgG. For peptide mass fingerprinting and subsequent analysis, selected gel bands were sliced and subjected to an in-gel protocol containing the reduction and alkylation with iodoacetamide [15].

### nanoLC fractionation

The digested peptide mixture of each gel band was separated by a prominence nano-liquid chromatography system (Shimadzu, Kyoto, Japan) with a flow rate ranging from 1 nL/min to 5 µL/min (Fig. S1). The sample was firstly loaded onto the trap column L-column 2 ODS (0.3×1 mm, 5 µm) (CERI's, Tokyo, Japan) at a flow rate of 0.05 mL/min with mobile phase C (water, 0.1% FA) for 5 min, then the trapped samples were separated in a MonoCap C-18 column (0.1×150 mm; GL. Sciences, Tokyo, Japan). The HPLC gradient was 5%–70% mobile phase B (acetonitrile/water, 95/5, 0.1% FA) in mobile phase A (acetonitrile/water, 5/95, 0.1% FA) at a flow rate of 2 µL/min for 90 min [16].

### LCMS-IT-TOF acquisition and data analysis

Coupled to nanoLC fractionation, IT-TOF-MS is equipped with an electrospray ionization (ESI) source in positive ion mode at a mass resolution of 40,000 FWHM. Accurate masses were corrected by calibration using the trifluoroacetic acid sodium solution (2.5 mM from 50 Da to 1000 Da as an external reference. The scan range was 50–1000 m/z. The voltage of ESI source and detector was 1.50 kV and 1.65 kV, respectively. The ion source temperature was kept at 230°C. The skimmer voltage was 8.5 V. The pressure of TOF region and ion trap was 1.4×10^4^ and 2.8×10^2^ Pa, respectively. The flow rate of trap cooling gas (Ar) and collision gas (Ar) was 94 and 43 mL/min, respectively. The ion accumulation time was 10 ms. The selected width of precursor ion was 3.0 m/z; the selected time and the collision-induced dissociation (CID) collision time were 20 ms and 50 ms, respectively. The collision energy was 50%. The resulting data was submitted onto the Mascot search engine (http://www.matrixscience.com) where a search was performed on the SwissProt database to determine candidate peptides. Mass tolerance for the precursor ion was set to 50 ppm, and the one for fragment ions was set to 0.2 Da. Meanwhile, fixed modification was set to carbamidomethyl (C); and variable modification was set to oxidation (M). For a standard peptide mixture, the mascot ion score should be more than 13 (p<0.05) and for a peptide mixture in complex samples, the mascot ion score should be more than 20 (p<0.01) [17], [18].

## Results

### nanoLC-IT-TOF MS analysis of BSA tryptic digests

BSA tryptic digested peptide mixture was performed on nanoLC fractionation, which was then subjected to LCMS-IT-TOF MS and MS/MS analysis. A total of 22 peptides were correctly identified and sequenced with high statistical significance (p<0.05), and the sequence coverage was up to 43% (Table S1). LC-MS or MS/MS methodologies are robust but reproducible experiments depend greatly on the performance of chromotographical separation. Compared with the BSA results using on-line nanoLC-IT-TOF, the BSA results using IT-TOF without nanoLC separation showed lower sequence coverage and lower statistical significance. Liquid chromatography directly determines the complexity of samples delivered to the mass spectrometer, and this complexity affects the selection of precursor ions required for MSn analysis. Thus, nanoLC with high performance was applied to the isolation of peptide mixtures that were subjected to LCMS-IT-TOF analysis, because nano-scaled analysis increases the sensitivity with extremely low sample volume.

### Gel electrophoresis of the serum β-catenin complex

**Figure 1:**
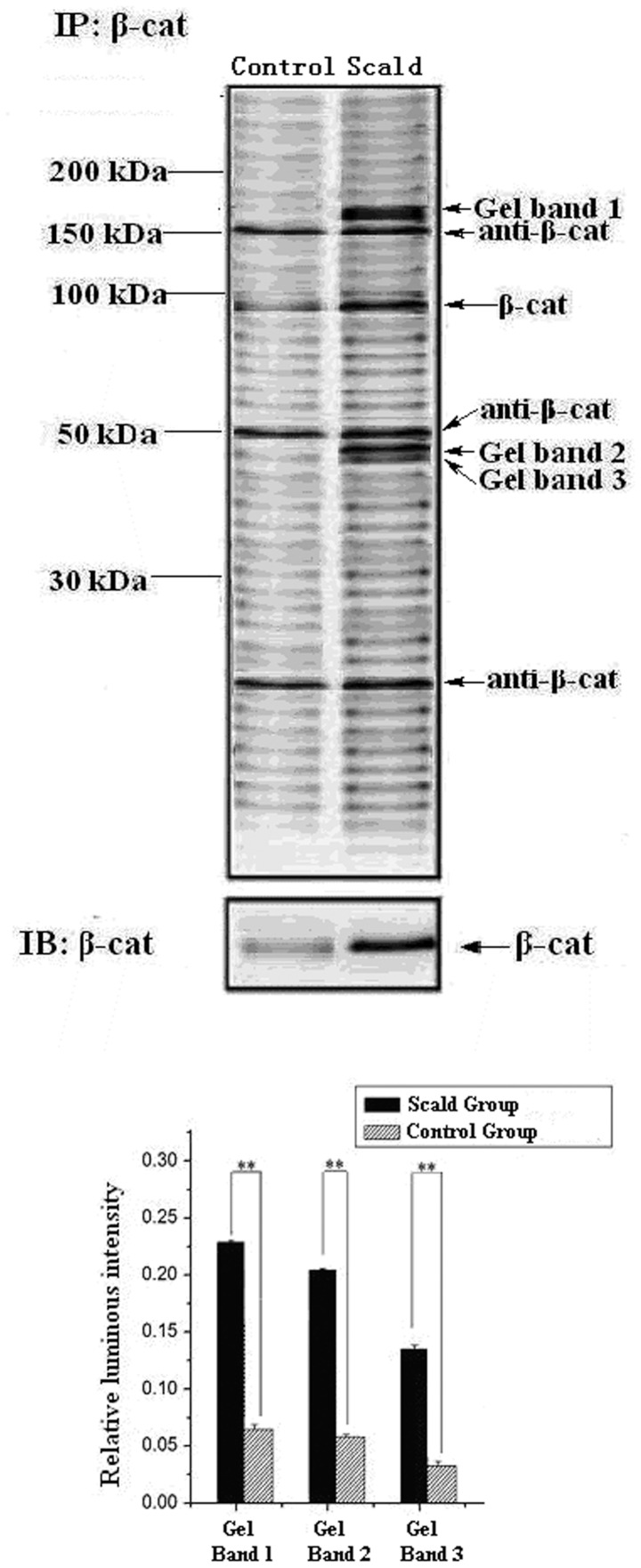
Up: Gel electrophoresis results of Gel Band 1, Gel Band 2 and Gel Band 3 representing the respective protein expressions (Scald Group vs. Control Group), anti-β-catenin IgG (molecular weight 150 kDa, heavy chain 50 kDa, light chain 25 kDa); Bottom: Relative luminous intensities of Gel Band 1, Gel Band 2 and Gel Band 3 representing the respective protein expressions (Scald Group vs. Control Group). Data are shown as mean ± S.D. of ten independent experiments (**p<0.01).

The serum sample was subjected to immunoprecipitation using anti-β-catenin antibodies. Next, SDS-PAGE was performed for isolation of the serum β-catenin complex. Contrasted with the control group, three clear gel bands with higher protein expression (p<0.01) in the scald group were selected for further analysis (Fig. 1), except for the gel bands containing β-catenin protein (molecular weight 92 kDa), and anti-β-catenin IgG (molecular weight 150 kDa, heavy chain 50 kDa, light chain 25 kDa) [19]. Meanwhile, β-catenin protein expression was validated by Western blotting.

### nanoLC-IT-TOF MS analysis of serum β-catenin complex

When the separation of digested peptide mixtures was completed, individual peptide ions were extracted from the peptide mixture and were subjected to CID following the mass analysis of resultant fragment ions. Both MS and MS/MS results were used for peptide identification through database searching because MS measurements alone were not sufficient for protein identification when dealing with complex mixtures. A total of 31 peptides were identified and sequenced with high statistical significance (p<0.01). A total of 3 proteins were identified and validated with high reliability and high reproducibility. Alpha-2-marcoglobulin (167 kDa, with sequence coverage up to 9%), serine protease inhibitor A3K (47 kDa, with sequence coverage up to 36%) and serine protease inhibitor A1A (46 kDa, with sequence coverage up to 34%) might complex with serum β-catenin in mice scald model (Table 1). Meanwhile, this experiment from immunoprecipitation to nanoLC-IT-TOF MS analysis was repeated in the other samples, and the results yielded the same protein identification, which were also identical to the gel electrophoresis results.

**Table 1:** Mascot search results for Gel Band 1, Gel Band 2 and Gel Band 3 tryptic digested peptides (p<0.01).

Source (Gel Band 1):	SPA3K_ Mouse				
Peptide sequence	Mr (expt)	Mr (calc)	Position	Modifications	Mascot ion score
**TMEEILEGLK**	1161.60	1161.60	94–103		36
**DLQILAEFHEK**	1341.68	1341.69	145–155		30
**ALYQTEAFTADFQQPTEAK**	2157.98	2158.02	158–176		40
**ELISELDER**	1102.55	1102.55	193–201		72
**ISFDPQDTFESEFYLDEKR**	2365.08	2365.08	218–236		27
**MQQVEASLQPETLR**	1644.81	1644.81	283–296	Oxidation (M)	39
**TLFPSQIEELNLPK**	1627.88	1627.88	301–314		23
**LEEDVLPEMGIK**	1387.67	1387.69	323–334	Oxidation (M)	56
**EVFTEQADLSGITETKK**	1894.91	1894.95	335–351		80
**AVLDVAETGTEAAAATGVIGGIRK**	2269.19	2269.22	361–384		23

Source (Gel Band 2):	A2M_ Mouse			
**AINYLISGYQR**	1296.66	1296.68	1016–1026	28
**AFAQAQSHIFIEK**	1488.76	1488.77	1061–1073	22
**ENGCFQQSGYLLNNAMK**	1915.83	1915.86	1089–1105	40
**ALLAYAFALAGNKAKR**	1676.93	1676.97	1160–1175	58
**ALSFYQPR**	980.51	980.51	1206–1213	30
**QQNSHGGFSSTQDTVVALQALSK**	2402.15	2402.18	1259–1281	36
**LLLQEVR**	869.53	869.53	1316–1322	32
**YNILPVADGK**	1088.59	1088.59	1348–1357	20
**MVSGFIPMKPSVKK**	1547.85	1547.86	1404–1418	44
**LTNQTLGFSFAVEQDIPVKNLK**	2461.30	2461.32	1441–1462	35

Source (Gel Band 3):	A1AT1_ Mouse				
**NHYQAEVFSVNFAESEEAKK**	2326.08	2326.09	154–173		50
**VINDFVEKGTQGK**	1433.73	1433.75	174–186		53
**KLDQDTVFALANYILFK**	1998.03	1998.08	193–209		26
**PFDPENTEEAEFHVDESTTVK**	2420.06	2420.07	216–235		42
**MQHLEQTLSKELISK**	1783.92	1783.95	278–292		54
**LAQIHFPR**	980.56	980.56	301–308		60
**LSISGEYNLK**	1122.59	1122.59	309–318		58
**TLMSPLGITR**	1087.61	1087.61	319–328		23
**TLMSPLGITR**	1087.61	1087.61	319–328	Oxidation (M)	73
**IFNNGADLSGITEENPLK**	1930.94	1930.96	329–347		33
**VVDPTHK**	794.43	794.43	407–413		67

## Discussion

Here we report on the first successful combination of immunoprecipitation, gel electrophoresis, nanoLC fractionation and LCMS-IT-TOF for a sensitive and rapid identification of serum β-catenin complex in mouse scald model. In this paper, three unknown binding partners with higher expression were acquired for MS analysis. A total of 31 peptides were identified and sequenced with high mass accuracy and high statistically significance. These three proteins were identified and validated with high reliability and high reproducibility. Alpha-2-marcoglobulin, serine protease inhibitor A3K and serine protease inhibitor A1A might have formed a complex with serum β-catenin in mice scald model. β-catenin is mainly degraded through the ubiquitin-proteasomal pathway [20], [21], [22]. However, there is another type of β-catenin degradation that involves protease [23]. The Omori group observed that β-catenin degradation in epidermal cells was realized through a protease-dependent mechanism, which led to the disruption of cell adhesion [24]. The Alman group demonstrated that β-catenin protein levels were elevated during the proliferative phase of wound healing, being expressed primarily in dermal mesenchymal cells, where it was transcriptionally active [25], [26], [27].

**Figure 2:**
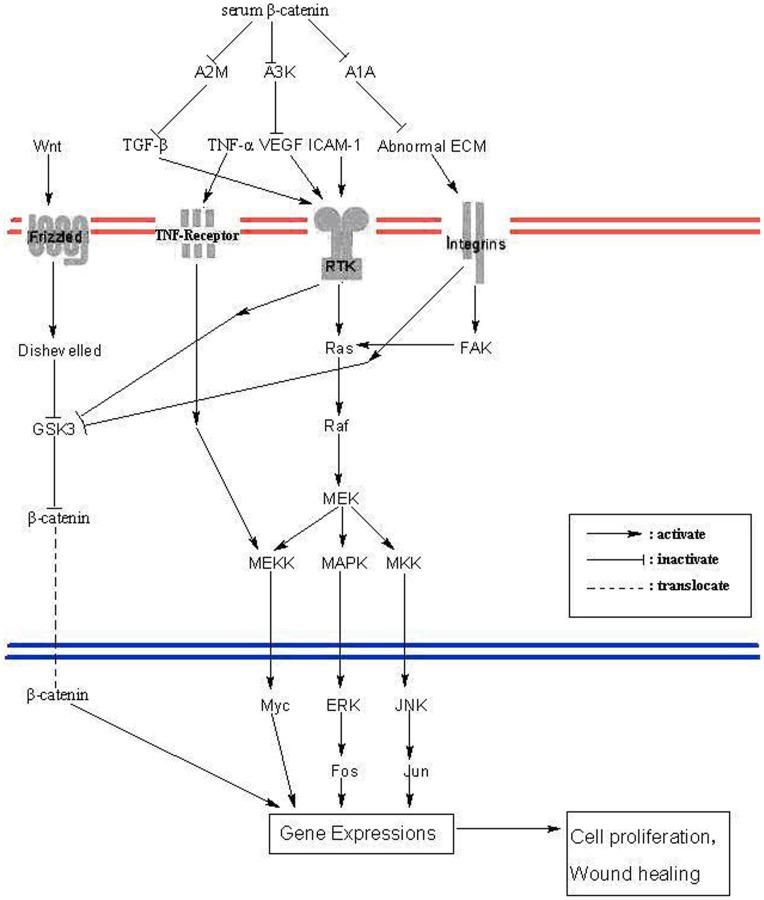
The putative serum β-catenin complex in scald model: alpha-2-marcoglobulin (A2M), serene protease inhibitor A3K (A3K) and serene protease inhibitor A1A (A1A).

With the help of raft-associated membrane protein caveolin-1, β-catenin was reported to be excreted into the extracellular milieu through exosome/prostasome associated pathways. Interference with the endocytic pathway using wortmannin did not inhibit prostasome excretion, but β-catenin overexpression promoted the extracellular accumulation of caveolin-1, which was helpful to β-catenin excretion. Meanwhile, β-catenin and caveolin-1 were both detected in cell-free human voided urine prostasomes [8]. Thus, this might be negative feedback towards the β-catenin related Wnt signaling, which would decrease the cell proliferation rate. (1) Alpha 2 macroglobulin often functions as an inhibitor of both fibrinolysis by inhibiting plasmin or kallikrein, and coagulation by inhibiting thrombin. More importantly, alpha 2-macroglobulin can act as a neutralizer of TGF-β [28]. (2) Ma et al. discovered that SERPINA3K blocked the overexpression of proinflammatory factors, such as VEGF, TNF-α, and ICAM-1 in the retina of OIR models and in cultured retinal cells exposed to hypoxia. VEGF was downregulated by SERPINA3K transcriptionally. Knockdown of SERPINA3K by siRNA resulted in the overexpression of VEGF and TNF-α in cultured retinal cells [29]. (3) Similarly, SERPINA1A is also an anti-inflammatory factor, because it could protect tissues from enzymes of inflammatory cells, especially neutrophil elastase which breaks down the elastin (an important component of ECM) to cause abnormal ECM [30]. Evidence has shown that abnormal ECM is able to result in the activation of Wnt and MAPKs signaling, where a recent study indicated that the activation of Wnt and MAPKs signaling protected against experimental emphysema in mice [31]. All three proteins play the role of limiting the damage caused by burn shock, but their target proteins contribute to the activation of Wnt and MAPKs signaling [32], [33], [34], [35], which then increases wound healing resulting from burn shock. Thus, it was suspected that the interaction of serum β-catenin with these partners might restrict their inhibitory effects on growth factors and proinflammatory factors so as to release these factors for the activation of Wnt and MAPKs signaling, which finally lead to the increased cell proliferation during wound healing (Fig. 2).

The methods of studying the protein complex were divided into two trends: traditional biochemical analysis and current MS analysis. (1) Traditional biochemical analysis included immunoprecipitation plus Western blot. When the precipitated protein complex was separated on a protein gel, primary Abs were added to detect the related proteins. However, this method has two bottlenecks: it is restricted to validate the existence of anticipated binding partners rather than identifying unknown binding partners, even though it is anticipated that there would be some proteins in the complex. Also, it is extremely difficult to acquire related Abs because the production of Abs requires sufficient amount of pure Ags. (2) Current MS analysis is immunoprecipitation coupled to MS directly. When the precipitated protein complex was separated on protein gel, in-gel tryptic digested peptides were manually desalted and then analyzed by MS. Compared with the BSA results using on-line nanoLC-IT-TOF (Table S1), the BSA results using IT-TOF without nanoLC separation showed low sequence coverage which was due to the ion suppression that low abundant peptides were masked by high abundant peptides and low mascot scores, which was due to insufficient MSn analysis. In addition, before the samples were loaded onto mass spectrometer, they often suffered from the manual desalting step, which also caused peptide loss. However, under the introduction of nano LC separation, the sequence coverage increased, which was especially important in the identification of PTMs (post translational modifications) because those modified peptides with low abundance were not masked due to sufficient LC separation. The mascot scores also increased a lot, which caused the low false positive rates. Similarly, when the tryptic digests of the protein complex were analyzed by nanoLC-IT-TOF without protein gel separation, it also brought about the low sequence coverage and low mascot scores due to ion suppressions resulting from the high abundant Abs required for immunoprecipitation. Moreover, IT-TOF integrates the perfect ion trap selection of precursor ions with the high mass precisions acquired by TOF. Thus, the combination of protein gel separation, nanoLC isolation and IT-TOF measurement demonstrating high sequence coverage with high statistical significance, was adopted in this paper, which was unprecedentedly adequate for sensitive and automated identification of the *in vivo* protein complex.

In conclusion, our study demonstrated that the on-line coupling of nano-LC with a LCMS-IT-TOF mass spectrometer was capable of sensitive and automated characterization of the serum β-Catenin complex in mice scald model. More importantly, this method would contribute to investigating more *in vivo* protein complexes that are very significant in clinical research.

## Supporting Information

Figure S1 Operating principle of nano-liquid chromatography system. The sample injected with the auto sampler is concentrated in the trap column. Then the flow channel selection valve is switched to elute the sample from the trap column. The sample is then separated in the reversed-phase column and introduced to the detector/mass spectrometer.
(TIF)

Table S1 Up: Mascot search results for BSA tryptic digested peptides (p<0.05) using on-line nanoLC-IT-TOF; Bottom: Mascot search results for BSA tryptic digested peptides (p<0.05) using IT-TOF without nanoLC isolation.
(DOC)
