# Supplementary figures and images for: Application of On-Line nanoLC-IT-TOF in the Identification of Serum β-Catenin Complex in Mice Scald Model
Source: PLoS One. 2012 Oct 9;7(10):e46530. doi: 10.1371/journal.pone.0046530 (PMC3467219; doi:10.1371/journal.pone.0046530)

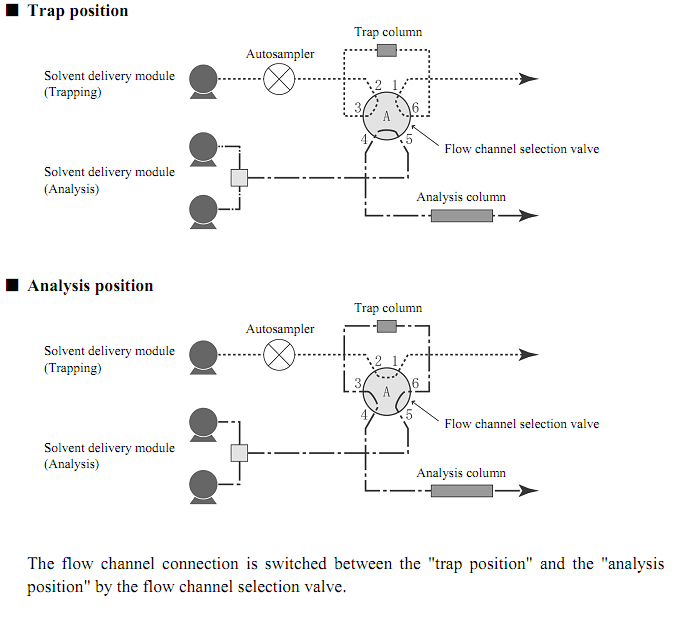

Supplement: Figure S1 — Operating principle of nano-liquid chromatography system. The sample injected with the auto sampler is concentrated in the trap column. Then the flow channel selection valve is switched to elute the sample from the trap column. The sample is then separated in the reversed-phase column and introduced to the detector/mass spectrometer. (TIF) [file pone.0046530.s001.tif]
