# Supplementary material for: Application of On-Line nanoLC-IT-TOF in the Identification of Serum β-Catenin Complex in Mice Scald Model
Source: PLoS One. 2012 Oct 9;7(10):e46530. doi: 10.1371/journal.pone.0046530 (PMC3467219; doi:10.1371/journal.pone.0046530)
Supplement: Table S1 — Up: Mascot search results for BSA tryptic digested peptides (p<0.05) using on-line nanoLC-IT-TOF; Bottom: Mascot search results for BSA tryptic digested peptides (p<0.05) using IT-TOF without nanoLC isolation. (DOC) [file pone.0046530.s002.doc]

**Table S1**

**on-line nanoLC-IT-TOF**:

| **Source:** | **ALBU_ Bovine** |  |  |  |  |
| --- | --- | --- | --- | --- | --- |
| **Peptide sequence** | **Mr (expt)** | **Mr (calc)** | **Position** | **Modifications** | **Mascot ion score** |
| **FKDLGEEHFK** | 1248.61 | 1248.61 | 35-44 |  | 16 |
| **LVNELTEFAK** | 1162.62 | 1162.62 | 66-75 |  | 59 |
| **TCVADESHAGCEK** | 1464.55 | 1464.55 | 76-88 |  | 10 |
| **SLHTLFGDELCK** | 1419.66 | 1419.67 | 89-100 |  | 35 |
| **NECFLSHKDDSPDLPK** | 1901.84 | 1901.85 | 123-138 |  | 35 |
| **LKPDPNTLCDEFK** | 1576.74 | 1576.74 | 139-151 |  | 31 |
| **YLYEIAR** | 926.48 | 926.49 | 161-167 |  | 38 |
| **AEFVEVTK** | 921.48 | 921.48 | 249-256 |  | 27 |
| **YICDNQDTISSK** | 1443.61 | 1443.62 | 286-297 |  | 89 |
| **LKECCDKPLLEK** | 1533.73 | 1533.74 | 298-309 |  | 24 |
| **RHPEYAVSVLLR** | 1438.80 | 1438.80 | 360-371 |  | 79 |
| **EYEATLEECCAK** | 1503.57 | 1503.57 | 375-386 |  | 50 |
| **DDPHACYSTVFDKLK** | 1795.80 | 1795.81 | 387-401 |  | 13 |
| **HLVDEPQNLIK** | 1304.70 | 1304.71 | 402-412 |  | 40 |
| **LGEYGFQNALIVR** | 1478.78 | 1478.79 | 421-433 |  | 90 |
| **KVPQVSTPLVEVSR** | 1638.93 | 1638.93 | 437-451 |  | 67 |
| **CCTESLVNR** | 1139.46 | 1139.46 | 499-507 |  | 63 |
| **RPCFSALTPDETYVPK** | 1880.89 | 1880.90 | 508-523 |  | 46 |
| **LFTFHADICTLPDTEK** | 1907.89 | 1907.90 | 529-544 |  | 17 |
| **KQTALVELLK** | 1141.71 | 1141.71 | 548-557 |  | 37 |
| **EACFAVEGPK** | 1107.49 | 1107.49 | 588-597 |  | 28 |
| **LVVSTQALA** | 1101.57 | 1101.58 | 598-607 |  | 22 |

**IT-TOF without nanoLC isolation:**

| **Source:** | **ALBU_ Bovine** |  |  |  |  |
| --- | --- | --- | --- | --- | --- |
| **Peptide sequence** | **Mr (expt)** | **Mr (calc)** | **Position** | **Modifications** | **Mascot ion score** |
| **LVNELTEFAK** | 1162.62 | 1162.63 | 66-75 |  | 18 |
| **YICDNQDTISSK** | 1443.61 | 1443.61 | 286-297 |  | 19 |
| **RHPEYAVSVLLR** | 1438.80 | 1438.81 | 360-371 |  | 19 |
| **EYEATLEECCAK** | 1503.57 | 1503.58 | 375-386 |  | 20 |
| **LGEYGFQNALIVR** | 1478.78 | 1478.77 | 421-433 |  | 20 |
| **KVPQVSTPLVEVSR** | 1638.93 | 1638.94 | 437-451 |  | 17 |
| **CCTESLVNR** | 1139.46 | 1139.47 | 499-507 |  | 13 |
| **RPCFSALTPDETYVPK** | 1880.89 | 1880.91 | 508-523 |  | 16 |
